# An Event-Related Potential Study of Social Information Processing in Adolescents

**DOI:** 10.1371/journal.pone.0154459

**Published:** 2016-05-18

**Authors:** Danielle diFilipo, Jillian Grose-Fifer

**Affiliations:** 1 Department of Psychology, The Graduate Center, City University of New York, New York City, New York, United States of America; 2 Department of Psychology, John Jay College of Criminal Justice, City University of New York, New York City, New York, United States of America; Vanderbilt University, UNITED STATES

## Abstract

Increased social awareness is a hallmark of adolescence. The primary aim of this event-related potential study was to investigate whether adolescents, in comparison to adults, would show relatively enhanced early neural processing of complex pictures containing socially-relevant information. A secondary aim was to investigate whether there are also gender and age differences in the ways adolescents and adults process social and nonsocial information. We recorded EEGs from 12–17 year-olds and 25–37 year-olds (*N* = 59) while they viewed pleasant pictures from the International Affective Picture System. We found age-related amplitude differences in the N1 and the LPP, and gender-related differences in the N2 region for socially-relevant stimuli. Social pictures (featuring mostly young children and adults) elicited larger N1s than nonsocial stimuli in adolescents, but not adults, whereas larger LPPs to social stimuli were seen in adults, but not adolescents. Furthermore, in general, males (regardless of age) showed larger N2s to nonsocial than to social images, but females did not. Our results imply that compared to adults, adolescents show relatively greater initial orientation toward social than toward nonsocial stimuli.

## Introduction

As social creatures, humans are wired to pay attention to other people. During adolescence there is a marked growth in the number and complexity of social relationships, particularly among peers [1], and so it becomes increasingly important for adolescents to accurately read social cues and respond accordingly. Much of the neurobiological research on social cue processing during adolescence has focused on developmental changes in face processing [2–4]. A preference for faces and face-like objects has been shown to be present soon after birth [5], and the ability to discriminate between emotional expressions begins to emerge as early as four months of age [6]. However, the abilities to recognize different people and to differentiate between facial expressions continue to develop throughout childhood and adolescence [3,6–11]. Other, more complex social skills are also still developing during adolescence, including the abilities to imagine another person’s viewpoint and to successfully evaluate interactions with others [2,3,12,13].

Perhaps unsurprisingly, adolescence appears to be a period of heightened sensitivity to social cues, which has frequently been explained by the relatively earlier maturation of the limbic system relative to cortical control areas [14,15]. In comparison to adults, adolescents are more likely to be distracted by irrelevant affective faces [16–19], experience greater emotional distress after social rejection [20,21], and are more likely to engage in risky behavior in the presence of peers [14,22].

There is also evidence to suggest that females, but not males, process social information preferentially over nonsocial information. In support of this, women have been shown to attend more to people than objects, whereas the reverse pattern has been found in men [23]. Women were found to have better recall about people’s physical appearances than men [24] and were more effective at extracting the gist about social information from a rapidly presented scene than men [25]. Women were also found to exhibit enhanced neural processing of complex social scenes in comparison to nonsocial scenes, whereas men showed a trend for the opposite phenomenon [26,27]. Currently, it is not clear whether there are also gender differences in social information processing in adolescents. But there is some evidence to suggest that female adolescents may be more sensitive to social cues than male adolescents. In a meta-analysis of over 50 studies, McClure [11] reported that even from infancy, females were better than males at identifying facial expressions. Also, social anxiety, which shows a steep increase in prevalence during adolescence, affects females more than males [28]. Therefore, it seems reasonable to expect adolescent females to be more attentive to social information than their male counterparts. One of the aims of our study was to test this hypothesis.

In our study, we used event-related potential (ERP) recording to examine the time course of information processing in adolescents and adults when viewing pleasant pictures of social and nonsocial scenes. The high temporal resolution of ERP recording allowed us to compare age and gender differences in both early, relatively automatic activity and later, more controlled neural cognitive processes [29]. Although in general, adolescents exhibit greater limbic system activation than adults in response to multiple different types of appetitive stimuli [30], it is unknown whether social stimuli also produce relatively *more* neural activation than nonsocial appetitive stimuli in adolescents compared to adults. Our study was designed to answer this question.

We based our ERP paradigm on a previous study by Proverbio et al. [27], who investigated gender differences in ERPs elicited by social and nonsocial stimuli in adults. In our study, we recorded participants’ EEGs while they viewed many of the same pleasant social and nonsocial scenes from the International Affective Picture System (IAPS) [31] that Proverbio and colleagues [27] used in their study. The IAPS stimuli have been widely used to study the effects of emotion and have been normed for use in both adults and adolescents.

We had three main hypotheses. First, previous research has shown that adolescents have a heightened sensitivity to social cues compared to adults and find it particularly difficult to ignore rapidly-presented emotional faces, even when they are not relevant to the task [16–19]. Therefore, we hypothesized that in comparison to adults, adolescents would show greater initial orienting to social than to nonsocial pictures. We tested this hypothesis by measuring two early ERP components (the posterior N1 and frontocentral N2), both of which have been shown to increase in amplitude with greater attentional focus [29,32] and when viewing pictures featuring humans [26,27]. The N1 and N2 are thought to reflect relatively automatic orienting processes [26,27,29,32]. Therefore, we predicted that social pictures would elicit disproportionately larger (more negative) N1 and N2 components than nonsocial pictures in adolescents, compared to adults.

Second, research suggests that females often show enhanced social information processing in comparison to men [11,23–28]. Because Proverbio and colleagues [26,27] showed that women had enhanced early brain activity (N2 responses) for social compared to nonsocial stimuli, whereas men tended to show the opposite pattern, we hypothesized that we would replicate this finding. We also assumed that we would find this effect regardless of age because others have shown that there is a female advantage for facial expression recognition from a very early age [11].

Third, the IAPS social stimuli predominantly feature pictures of younger children or adults (rather than adolescents), and there is evidence suggesting that humans preferentially attend to pictures of people of their own age [33]. Because of this, we hypothesized that for adults, the social stimuli would continue to hold their attention more effectively than the nonsocial stimuli, but the reverse would be true for adolescents. A relatively late ERP component, the late positive potential (LPP), has been shown to increase in amplitude when stimuli are perceived to be particularly motivationally salient [34–37]. Therefore, we predicted that nonsocial pictures would elicit larger or similarly sized LPPs to those elicited by social stimuli in adolescents. In contrast, we predicted that social images would elicit larger LPPs than nonsocial pictures in adults.

## Method

### Participants

This research was approved by the City University of New York UI IRB Board, protocol # 302127. A total of 85 participants were recruited from the community through flyers posted at local high schools, colleges, and in the community, and through online postings at Craigslist.com. All participants had normal or corrected-to-normal vision and reported that they had no history of neurological or psychiatric disorders. Adult participants provided written informed consent, while adolescents under the age of 18 provided written parental consent and gave written assent. Participants were compensated with $25 for their time. The data for 26 participants (19 adolescents and 7 adults) were excluded. Of these excluded participants, 25 had an insufficient number of acceptable trials largely because of movement artifact, and one had very low accuracy on the ERP task. The remaining participants consisted of 30 adolescents (12 males) between the ages of 12–17 years (*M* = 15.5 years, *SD* = 1.5 years) and 29 adults (15 males) ages 25–37 years old (*M* = 29.5 years, *SD* = 3.5 years). Among the adolescents, 46.7% identified as Hispanic, 20% as Asian, 13.3% as White, 10% as Black, and 10% identified as another race. Among the adults, 31% identified as Hispanic, 3.4% as Asian, 24.1% as White, 20.7% as Black, and 20.8% identified as another race. Handedness was assessed using the Edinburgh Handedness questionnaire [38]; of the 30 adolescents, 25 were right-handed (*M* = 0.70, *SD* = 0.25), and the remainder were left-handed (*M* = -0.61, *SD* = 0.20). Of the 29 adults, 26 were right-handed (*M* = 0.79, *SD* = 0.25) and the remainder were left-handed (*M* = -0.53, *SD* = 0.31). The mean level of education for the adults ranged from 13 to 20 years (*M* = 15.3 years, *SD* = 1.8 years).

### Materials and Procedure

#### EEG recording

During the ERP task, EEG was recorded from 62 sintered silver/silver chloride scalp electrode sites using an elasticized Neuroscan Quikcap electrode cap, Neuroscan Synamps RT amplifier, and Neuroscan 4.4 Acquire software (Compumedics, El Paso, TX), with a midline central reference electrode between Cz and CPz. All electrode impedances were below 5 kΩ. The EEG was recorded continuously with a bandpass of 0.01 to 200 Hz at a rate of 1000 Hz, and analyses were conducted off-line with Neuroscan SCAN 4.4 Edit software.

#### ERP stimuli

We used 160 pleasant, color photographs from the IAPS [31] as ERP stimuli. We restricted the stimuli to pleasant images because we wanted to use images that were similar to those in a study by Proverbio et al. [27] who found gender-related differences in social information processing in adults, and also because we did not want to distress the adolescents by showing negative images. Stimuli were divided into two types: social and nonsocial. The social stimuli (*n* = 80) depicted one or more people of a variety of ages engaged in various activities. The nonsocial stimuli included photos of scenic landscapes (*n* = 35), objects (*n* = 12), food (*n* = 12), or animals (*n* = 21). The social and nonsocial stimuli were balanced in terms of valence (Social: *M* = 6.40, *SD* = 0.6; Nonsocial: *M* = 6.46, *SD* = 0.6) and arousal (Social: *M* = 4.1, *SD* = 0.9; Nonsocial: *M* = 4.3, *SD* = 0.9) according to the IAPS normed ratings [31]. Social and nonsocial stimuli did not differ significantly in terms of their mean Ojanpua contrast, mean luminance, mean spatial frequency [39], or complexity [40] (all *p*s > 0.1). To encourage participants to pay attention to the stimuli, we asked them to respond whenever they saw one of 10 easily identifiable target stimuli which featured images of sheep prominently displayed in the center of the photo. Targets were presented a total of 60 times throughout the experiment.

Each stimulus was displayed on a Dell 1908 Flat Panel LCD monitor for 1 second with an average inter-stimulus interval of between 2 and 4 seconds (mean = 3 seconds) using E-prime v.2.0 software (Psychology Software Tools, Inc.). The viewing distance was 67 cm and the image (15.5 cm x 15.5 cm) subtended a visual angle of 13 degrees by 13 degrees. Stimuli were presented in six blocks; each block contained equal numbers of social and nonsocial stimuli (i.e., 26 or 27 of each type). The first three blocks contained the same stimuli as the last three blocks; thus, each stimulus was shown twice to ensure that we had enough trials for the ERP averages. All images within a block were presented in a pseudo-random order so that social and nonsocial stimuli were interspersed fairly evenly and targets appeared unpredictably.

#### ERP task

Participants pressed the left mouse button with their dominant hand whenever they saw a target image. The purpose of the ERP task was simply to ensure that participants were paying attention to the stimuli; therefore, we did not analyze the ERPs to the target stimuli. No response was required to the social or nonsocial stimuli. This was because we did not want any ERP components evoked by motor processes to contaminate the components of interest in the study, i.e., those elicited by the social and nonsocial stimuli. Participants completed one practice session and were given the option of completing more practice sessions until they felt comfortable to proceed with the task proper.

#### Memory task

To investigate whether participants remembered social stimuli better than nonsocial stimuli, participants completed an unexpected memory test after the ERP task. Very little time (approximately 2 minutes) elapsed between the end of the ERP task and the memory test. During the memory test, the participant viewed 120 photos (60 social and 60 nonsocial). Half of all the pictures had been seen during the ERP task, while the others (foils) had not been seen before but had similar content to the ERP task pictures. Participants pressed the left mouse button if they had seen the picture before or pressed the right mouse button if the picture was new to them. Their accuracy on the task was recorded using the E-prime program.

#### ERP analysis

ERPs were extracted from the EEG offline. All data was re-referenced to averaged mastoids. Sweeps were sampled from 200 ms prior to stimulus onset until 1200 ms after stimulus onset. Epochs were bandpass filtered from 0.1 to 30 Hz and baseline corrected using the averaged EEG from 200 ms prior to stimulus onset. Epochs were corrected for eye movement artifacts using a regression procedure [41]. Sweeps were excluded where the EEG exceeded ±50 μV. The percentage of accepted trials ranged from 21 to 99% (*M* = 57%, *SD* = 20%) and did not differ significantly with age (Adolescents: 24–89%, *M* = 53%, *SD* = 25%; Adults: 21–99%, *M* = 59%, *SD* = 25%), gender (Females: 21–99%, *M* = 54%, *SD* = 25%; Males: 24–94%, *M* = 58%, *SD* = 27%), or stimulus type (Nonsocial: 21–99%, *M* = 56%, *SD* = 19%; Social: 24–94%, *M* = 57%, *SD* = 20%), all *p*s > .08.

The amplitudes of the ERP components were measured based on previous studies and visual inspection of the grand averages. The N1 was measured as the mean activity between 150 and 200 ms after stimulus onset at the electrodes at which it appeared largest in the grand averages, i.e., TP7/TP8, CP5/CP6, P5/P6, P7/P8 electrodes [42]. The N2 was measured as the mean activity averaged over a cluster of electrodes (FC1, FC2, FCZ, C1, C2, and CZ) between 210 and 270 ms [26,27]. The LPP was measured as the mean amplitude averaged over a cluster of electrodes (P1, PZ, P2, POZ, PO3, and PO4) in three successive 200 ms time windows at 400–600 ms, 600–800 ms, and 800–1000 ms after stimulus onset. We used these windows because the earliest LPP window is assumed to reflect more bottom-up attentional processes, whereas the later windows are posited to reflect more elaborative cognitive processing of stimuli [43]. For a similar approach see [43,44].

Mixed-design repeated-measures analyses of variance (ANOVAs) were run for each of the ERP components (i.e., N1, N2, LPP). Specific analyses for each component are described in the results section. Whenever assumptions of sphericity were violated, a Greenhouse-Geisser correction was applied, but uncorrected degrees of freedom are reported here for ease of interpretation. To investigate whether ERP amplitude predicted performance on the memory task, we performed simple bivariate correlations between the amplitude of the ERP components (N1, N2, and LPP in each window) and accuracy on the memory task.

## Results

### ERP Target Accuracy

As described earlier, one participant had very low accuracy scores on the target task (3.33%), indicating either a lack of attention or faulty recording of the responses, and so was excluded from the study. An ANOVA using within-subject factors of Stimulus (social, nonsocial) and between-subject factors of Age Group (adolescent, adult) and Gender (male, female) showed that the remaining participants had high accuracies (*M* = 98.8%, *SD* = 2.5%), indicating good attention to the task. There was no significant difference in accuracy between adults (*M* = 98.2%, *SD* = 3.4%) and adolescents (*M* = 99.4%, *SD* = 0.8%), or between males (*M* = 98.6%, *SD* = 3.1%) and females (*M* = 99.0%, *SD* = 1.9%), nor was there an interaction between Age Group and Gender for accuracy (*p*s > .07).

### Recognition Memory

An ANOVA using within-subjects factors of Stimulus (social, nonsocial) and between-subject factors of Age Group (2) and Gender (2) showed that overall, participants remembered social stimuli, i.e., pictures containing people, better (*M* = 82.4%, *SD* = 8.7%) than nonsocial stimuli, i.e., pictures without people in them (*M* = 68.2%, *SD* = 9.6%), *F*(1, 55) = 144.3, *p* < 0.001, η_p_^2^ = 0.72. There were no main effects of Age Group or Gender and no interaction between Age Group and Gender (all *p*s > 0.3).

The social stimuli used in the recognition test were equally subdivided into those containing younger people (under 25 years old, *n* = 40) and those containing older people (over 35 years old, *n* = 40). An ANOVA using within subject-factors of Stimulus Age (old, young) and between subject-factors of Age Group and Gender, revealed that overall, pictures containing older people (*M* = 83.9%, *SD* = 7.3%) were remembered better than pictures with younger people (*M* = 81.0%, *SD* = 11.5%), *F*(1, 55) = 6.8, *p* = 0.012, η_p_^2^ = 0.11. There were no main effects of Gender or Age Group and there was no interaction between Age Group and Gender (all *p*s > 0.3).

### ERP Data

**Fig 1:**
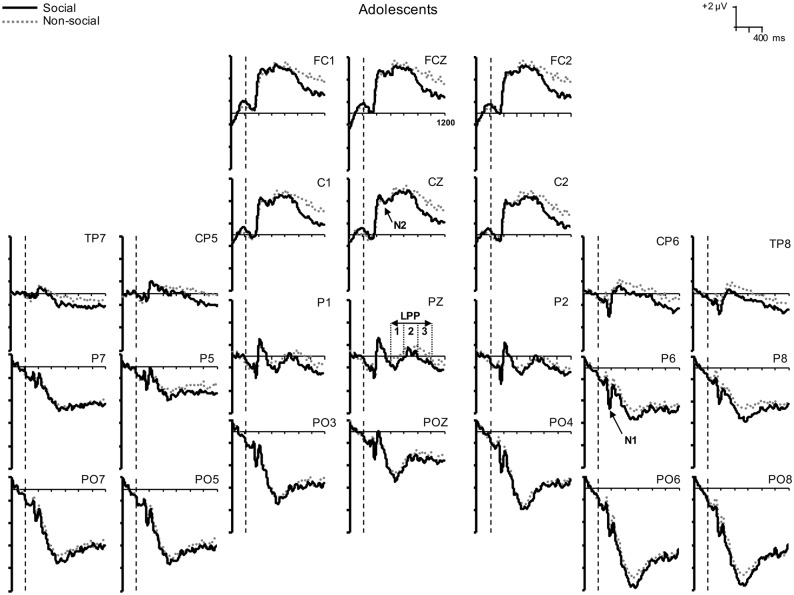
Adolescent grand average ERPs. Grand average ERPs at 24 electrodes elicited by social (solid line) and nonsocial (dotted line) stimuli in adolescent participants. Stimulus onset occurred at 0 ms as indicated by the vertical dashed lines.

**Fig 2:**
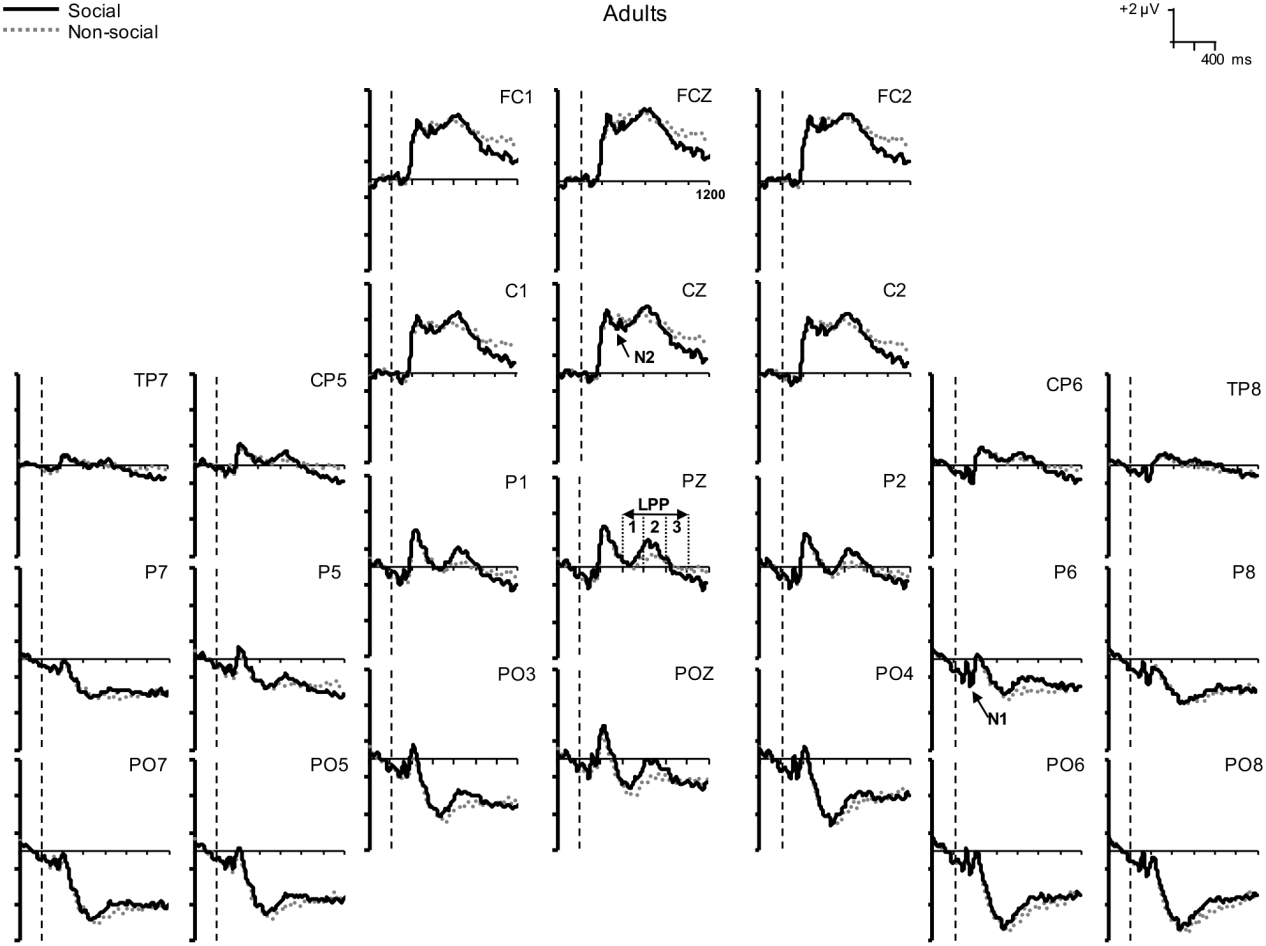
Adult grand average ERPS. Grand average ERPs at 24 electrodes elicited by social (solid line) and nonsocial (dotted line) stimuli in adult participants. Stimulus onset occurred at 0 ms as indicated by the vertical dashed lines.

**Fig 3:**
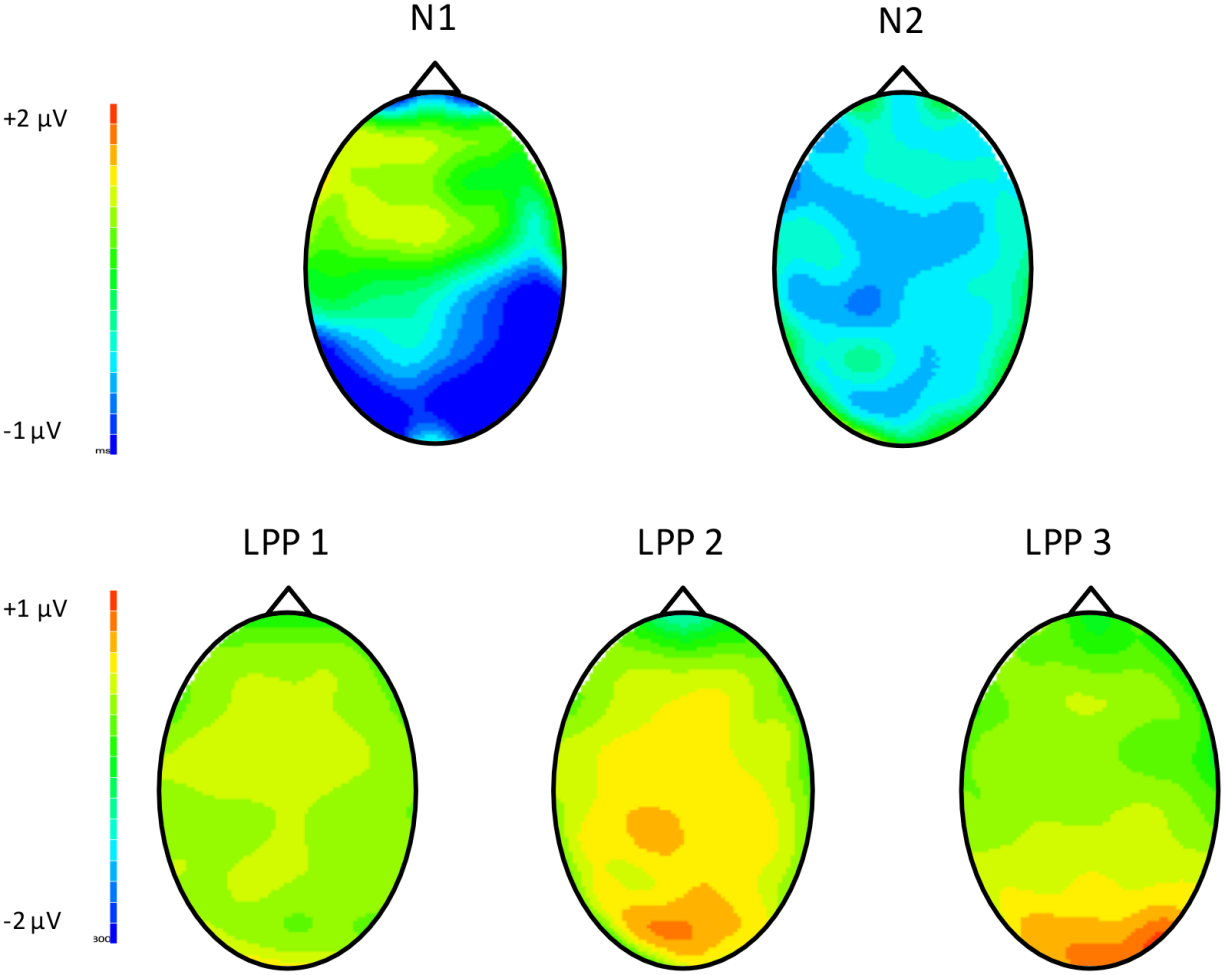
Headmaps for the ERP components. Data were collapsed across age group and gender.

The grand-averaged ERP waves for social and nonsocial stimuli are shown in Fig 1 for adolescents and Fig 2 for adults. The headmaps showing the topographical distribution of the ERP components of interest are shown in Fig 3. Mean amplitudes of the ERP components are shown in Table 1.

**Table 1:** Mean and (SEM) ERP amplitudes (μV) for social and nonsocial stimuli for each gender and age group.

ERP	Stimulus	Adolescents			Adults			All Ages	
		Female	Male	All	Female	Male	All	Female	Male
**N1**	*Social*	-2.46 (0.37)	-2.26 (0.46)	-2.36 (0.29)	-0.87 (0.42)	-0.69 (0.41)	-0.78 (0.29)	-1.66 (0.28)	-1.47 (0.31)
	*Nonsocial*	-2.01 (0.42)	-1.11 (0.51)	-1.56 (0.33)	-0.80 (0.48)	-0.91 (0.46)	-0.86 (0.33)	-1.40 (0.32)	-1.01 (0.34)
**N2**	*Social*	2.73 (0.57)	4.27 (0.70)	3.50 (0.45)	2.42 (0.65)	3.40 (0.63)	2.91 (0.45)	2.58 (0.43)	3.83 (0.47)
	*Nonsocial*	2.79 (0.48)	3.31 (0.58)	3.05 (0.38)	2.60 (0.54)	2.55 (0.52)	2.57 (0.38)	2.70 (0.36)	2.93 (0.39)
**LPP 1**	*Social*	-3.67 (0.93)	-2.49 (1.13)	-3.08 (0.73)	-1.31 (1.05)	-1.03 (1.02)	-1.17 (0.73)	-2.49 (0.70)	-1.76 (0.76)
	*Nonsocial*	-3.02 (0.83)	-2.42 (1.02)	-2.72 (0.66)	-1.37 (0.95)	-1.57 (0.91)	-1.47 (0.66)	-2.19 (0.63)	-2.00 (0.68)
**LPP 2**	*Social*	-2.72 (0.87)	-1.40 (1.07)	-2.19 (0.66)	-0.50 (0.99)	-0.18 (0.95)	-0.29 (0.66)	-1.61 (0.66)	-0.79 (0.72)
	*Nonsocial*	-2.05 (0.82)	-1.79 (1.01)	-1.95 (0.62)	-0.99 (0.93)	-0.95 (0.90)	-0.91 (0.62)	-1.52 (0.62)	-1.37 (0.68)
**LPP 3**	*Social*	-2.89 (0.81)	-1.72 (0.99)	-2.31 (0.64)	-1.28 (0.92)	-0.93 (0.89)	-1.10 (0.64)	-2.08 (0.61)	-1.33 (0.67)
	*Nonsocial*	-1.99 (0.78)	-1.62 (0.96)	-1.80 (0.62)	-1.02 (0.89)	-1.07 (0.86)	-1.04 (0.62)	-1.50 (0.59)	-1.34 (0.64)

LPP 1 = LPP in window 1; LPP 2 = LPP in window 2; LPP 3 = LPP in window 3.

#### N1

Visual inspection of the waveforms (Figs 1 and 2) and headmaps (Fig 3) indicated that the N1 was larger over the right hemisphere than over the left. Therefore, we performed an ANOVA that included Hemisphere (left: [mean of TP7, CP5, P7, P5], right: [mean of TP8, CP6, P8, P6]) and Stimulus (social, nonsocial) as within-subjects factors, and Gender (male, female) and Age Group (adult, adolescent) as between-subjects factors. Our observation that N1 amplitudes were considerably larger over the right hemisphere (*M* = -1.54 μV, *SE* = 0.21 μV) than the left (*M* = -0.56 μV, *SE* = 0.20 μV) was confirmed, *F*(1, 55) = 26.65, *p* < 0.001, η_p_^2^ = 0.33. Therefore, we restricted our N1 analysis to the right hemisphere data only. These data showed a Stimulus X Age Group interaction, *F*(1, 55) = 4.32, *p* = 0.042, η_p_^2^ = 0.07. ANOVAs were performed for adolescents and adults separately to explore this interaction further. Social stimuli were found to elicit larger N1s than nonsocial stimuli in adolescents, *F*(1, 28) = 8.71, *p* = 0.006, η_p_^2^ = 0.24, but not in adults, *F* < 1.

#### N2

The grand averaged data (Figs 1 and 2) show that the N2 (a negative-going wave) was superimposed on a large positivity with similar timing. Therefore, in Table 1, larger N2 amplitudes are reflected as smaller (relatively more negative) positivities. An ANOVA with between-subjects factors of Gender (2) and Age Group (2) and a within-subjects factor of Stimulus (social, nonsocial) revealed a Stimulus X Gender interaction, *F*(1, 55) = 5.24, *p* = 0.026, η_p_^2^ = 0.09. Similar ANOVAs performed for males and females separately revealed that nonsocial stimuli elicited larger (relatively more negative) N2 amplitudes than social stimuli in males, *F*(1, 25) = 13.41, *p* = 0.001, η_p_^2^ = 0.35, but not females, *F* < 1.

#### LPP

We first ran omnibus ANOVAs for each window of the LPP with Stimulus (social, nonsocial) as within-subjects factors and Age Group (2) and Gender (2) as between-subjects factors. None of these ANOVAs showed a main effect of Gender or any interactions with Gender (all *p*s > 0.14), so we reran the ANOVAs but omitted Gender from the between-subjects factors. These results are described below.

In window 1 (400–600 ms) of the LPP, there were no main effects of Age Group (*p* > 0.08) or Stimulus (*F* < 1), nor were there any Age Group X Stimulus interactions (*p*s > 0.11).

In the second (600–800 ms) LPP window, there was an interaction between Age Group and Stimulus, *F*(1, 57) = 4.02, *p* < 0.05, η_p_^2^ = 0.06. Adults showed larger (more positive) LPPs to social than nonsocial stimuli, *F*(1, 28) = 10.54, *p* = 0.003, η_p_^2^ = 0.27, whereas adolescents showed no significant difference in LPP amplitude between the social and nonsocial stimuli, *F* < 1. In the third (800–1000 ms) LPP window, there were no main effects of Age Group (*p* > 0.21) or Stimulus (*p* > 0.11), nor were there any Age Group X Stimulus interactions (*p*s > 0.18).

### ERPs and Recognition Memory Performance

The correlation between ERP amplitude and accuracy on the recognition memory task was significant for only one comparison: Larger (more negative) N1 amplitudes were associated with better memory for nonsocial stimuli, *r* (59) = -2.71, *p* = 0.038, all other *p*s > 0.1.

## Discussion

The results of this study supported most of our hypotheses. Our first hypothesis, that adolescents would show relatively greater early neural activity to socially-relevant stimuli than adults, was confirmed. In general, pleasant pictures (regardless of social content) elicited larger N1 amplitudes (over the right hemisphere) in adolescents than adults, which is consistent with the growing literature that suggests that adolescence is a period of heightened responsivity to affective cues in general [45]. Moreover, social stimuli elicited larger N1s than nonsocial images in adolescents, but not in adults. Numerous studies in adults have shown that increased N1 amplitudes are associated with greater attentional focus [29,32]. The topographical distribution of the N1 (larger over the right posterior-temporal hemisphere) supports the likelihood that for social stimuli, the N1 includes contributions from the face-specific N170 [46,47] and the body-specific N190 [48,49] components. Given the early timing of the N1 (150 to 210 ms), our findings lead us to the interpretation that for adolescents, social images draw initial attention more effectively than other motivationally salient stimuli. In contrast, adults did not seem to preferentially orient toward social compared to nonsocial images. This may seem surprising because it has been well documented that faces produce larger N170s than non-face stimuli in adults. However, our social stimuli were very different from the cropped faces typically used in N170 experiments, in that they featured people in more naturalistic settings. Within our social stimuli, about half were close-up shots, but faces were not clearly visible in 33 of the 80 pictures. Therefore, the N1 elicited by social pictures in our study is probably smaller than that elicited by traditional N170 face stimuli. Similarly, our nonsocial stimuli may have elicited larger N1s than in most N170 studies because we included pictures of animals, and in many of these their faces were clearly visible. Rousselet and colleagues [50] have reported that animal and human faces elicit similarly sized N170s, albeit at slightly different latencies. In previous studies of adults using similar stimuli and tasks to ours, two showed that social photos elicited larger N1 amplitudes than nonsocial ones [26,51], but another did not [27]. However, this N1 enhancement to social stimuli has been documented as being relatively small [26], which may be why it has only been found when using considerably larger stimulus sets [26,51] than in our study. All of these factors may have contributed to why we did not see a larger N1 for social compared to nonsocial images in adults. Nevertheless, we want to stress that we did find age-related differences in the N1 that suggest that adolescents show enhanced initial social information processing relative to adults.

Our second hypothesis, that females (regardless of age) would show enhanced processing of social relative to nonsocial information and that males would show the opposite pattern, was partially supported. We replicated the finding reported by Proverbio and colleagues [26,27] that males have larger N2s for nonsocial compared to social images in the 210 to 270 ms window. In contrast, we did not find evidence of a female advantage for social information processing in this same time window.

Finally, we had hypothesized that for adults, social stimuli would continue to hold their attention more effectively than the nonsocial stimuli, but the reverse would be true for adolescents. The LPP analysis in the 600 to 800 ms window supported this hypothesis; adults showed enhanced LPPs to social stimuli compared to nonsocial stimuli, whereas adolescents showed no stimulus-related differences. Using a similar paradigm, Proverbio and colleagues [26] also found that adults had larger LPPs for social compared to nonsocial IAPS images in a similar time window (they only measured the LPP from 500 to 700 ms, where it was maximal). We did not find evidence for this effect in earlier or later LPP windows. This could be interpreted as evidence that adults were consciously paying more attention to the social compared to nonsocial scenes because the earlier (400 to 600 ms) LPP window is more likely to reflect bottom-up processing than the later ones, which reflect progressively greater top-down influences [43]. This effect may have been relatively short-lived (i.e., did not extend to 800 to 1000 ms) because of the nature of the ERP task. Essentially, both social and nonsocial stimuli were non-targets and so participants, after showing a passing interest in the people in the scenes, may have decided that these pictures did not warrant further sustained attention. Alternatively, the effect may have been evident in this window simply because this was where the LPP was largest in general.

Although others have suggested that socially-relevant cues may be more salient than other types of appetitive cues during adolescence [52], to our knowledge, this is the first ERP study to empirically test this supposition. Initially, adolescents appear to preferentially orient toward social information more than adults, which is also consistent with studies that have shown that rapidly presented emotional faces capture attention more effectively in adolescents than in adults. Adolescents have been shown to be more distracted than adults by irrelevant, peripheral fearful faces in a flanker task [16] and by happy NoGo face stimuli in a Go/NoGo paradigm [19]. Furthermore, when Monk and colleagues asked participants to assess a non-emotional aspect (such as nose width) of an emotional face, adolescents showed greater activation in emotion-related brain areas than adults [18], indicating that adolescents found it difficult to ignore emotional features even when they were secondary to the task.

In our study we found that the adolescents’ early biases (as reflected in the N1) toward social stimuli appeared to be short-lived. Our LPP results show that in later time frames (> 400 ms), adolescents spent just as much neural effort processing the nonsocial photographs as they did the social photographs. The LPP is thought to reflect both sustained attention and enhanced cognitive processing in relation to a stimulus [29,36,53]. Only adults showed any evidence of larger LPPs to social relative to nonsocial stimuli. One possible interpretation of these data is that during the N1 time frame, adolescents are wired to automatically devote additional neural resources in order to extract important socially-relevant information from a scene. This idea is supported by studies which suggest that the N170 encodes social categories, at least in terms of gender-typicality [54], race [55], and in-group/out-group status [56]. Then, in later time frames (as reflected by activity in the LPP) this information may be used to decide whether to devote further cognitive resources to processing this information. Our LPP results suggest that adolescents found the social and nonsocial stimuli to be equally deserving of their sustained attention.

There are two potential reasons for why the social stimuli did not produce an enhanced LPP in adolescents relative to nonsocial stimuli. First, because the social stimuli that we used predominantly featured images of adults and younger prepubescent children (only 10 of the 80 social pictures depicted teenagers), the adolescents in our study may not have found them particularly motivationally salient. In a recent meta-analysis examining the phenomenon of the own-age bias on memory, Rhodes and Anastasi [33] found that children and adults tend to exhibit better memory for pictures of people their own age, suggesting that they direct their attention more to peers than to other age groups. However, this explanation is difficult to reconcile with the results from the unexpected memory test. We found that both adults and adolescents had better memory for social than for nonsocial pictures, and that both groups remembered the pictures of older people better than younger people. Although both age groups showed increased neural activation for social compared to nonsocial pictures (albeit in different time frames), with the exception that greater N1 amplitudes were predictive of better memory for nonsocial stimuli, we were unable to find any significant correlations between ERP amplitude and recognition memory. It is possible that the memory test was insensitive to age-related differences in social information processing because we only showed half of the ERP stimuli. We had decided to use this shortened form of the memory test because in pilot testing, some of our youngest participants complained that they were tired after the ERP session. An alternative explanation for the lack of LPP enhancement for social stimuli in adolescents is that the nonsocial stimuli, especially those that depicted food and animals, may have been particularly appetitive to the adolescents and so were just as effective in holding their attention as social stimuli. Unfortunately, we did not include any images of food in the memory test and there were relatively few images of animals, and so we were unable to test this empirically.

We found little evidence to support the idea that adolescent or adult females were predisposed to process social information preferentially over nonsocial information. In contrast, we found that males showed enhanced early neural processing of nonsocial compared to social information, and this phenomenon was present in adolescents as well as adults. Therefore, it is possible that males may be wired to find socially-salient information less attention-grabbing in comparison to other types of appetitive stimuli.

This study is not without its limitations. First, we chose our social and nonsocial stimuli according to the normed ratings for pleasantness and arousal, but we did not assess individual differences for arousal or valence ratings of the photographs. These measures may have been better predictors of the neural responses and may also have shed some light on what adolescents found to be particularly motivationally salient among the photographs. Secondly, because we did not use the entire stimulus set in the recognition memory test, we were unable to make reliable inferences about whether relatively larger N1s (in adolescents) and LPPs (in adults) for social stimuli affected the ability to encode this information successfully.

In summary, these data support the implication that during adolescence, there is a heightened initial orientation toward social cues. This orientation may be important for helping adolescents to rapidly extract key information from a social scene, which they then may use to determine whether further attention is warranted.

## Appendix

IAPS images

Social: 1340, 1601, 2019, 2020, 2036, 2056, 2091, 2102, 2158, 2211, 2214, 2216, 2222, 2224, 2270, 2273, 2300, 2302, 2303, 2304, 2306, 2308, 2342, 2345, 2346, 2362, 2370, 2372, 2373, 2374, 2377, 2381, 2382, 2385, 2389, 2390, 2392, 2395, 2411, 2442, 2485, 2495, 2500, 2501, 2506, 2510, 2511, 2513, 2518, 2521, 2530, 2550, 2580, 2600, 2870, 4532, 4537, 4542, 4603, 4610, 4612, 4614, 5410, 5836, 7660, 8120, 8121, 8130, 8180, 8300, 8312, 8330, 8350, 8380, 8420, 8460, 8461, 8490, 8496, 8497, 8499

Nonsocial: 1419, 1540, 1560, 1590, 1600, 1602, 1603, 1640, 1650, 1660, 1661, 1670, 1675, 1720, 1722, 1731, 1740, 1810, 1812, 1900, 1942, 1947, 5030, 5130, 5201, 5220, 5300, 5390, 5395, 5450, 5533, 5593, 5611, 5635, 5665, 5711, 5720, 5726, 5731, 5740, 5750, 5781, 5800, 5814, 5849, 5870, 5890, 5900, 5920, 5990, 5991, 5994, 7039, 7057, 7140, 7165, 7192, 7220, 7242, 7280, 7282, 7286, 7291, 7320, 7430, 7450, 7472, 7480, 7481, 7490, 7495, 7500, 7501, 7508, 7510, 7530, 7545, 7546, 8162, 8500
